# Identification and Validation of Hub Genes for Predicting Treatment Targets and Immune Landscape in Rheumatoid Arthritis

**DOI:** 10.1155/2022/8023779

**Published:** 2022-10-22

**Authors:** Xinling He, Ji Yin, Mingfang Yu, Haoyu Wang, Jiao Qiu, Aiyang Wang, Xueyi He, Xiao Wu

**Affiliations:** ^1^The Affiliated Traditional Chinese Medicine Hospital of Southwest Medical University, Luzhou 646000, China; ^2^The Traditional Chinese Medicine Hospital of Luzhou, Luzhou 646000, China

## Abstract

**Background:**

Rheumatoid arthritis (RA) is recognized as a chronic inflammatory disease featured by pathological synovial inflammation. Currently, the underlying pathophysiological mechanisms of RA remain unclear. In the study, we attempted to explore the underlying mechanisms of RA and provide potential targets for the therapy of RA via bioinformatics analysis.

**Methods:**

We downloaded four microarray datasets (GSE77298, GSE55235, GSE12021, and GSE55457) from the GEO database. Firstly, GSE77298 and GSE55457 were identified DEGs by the “limma” and “sva” packages of R software. Then, we performed GO, KEGG, and GSEA enrichment analyses to further analyze the function of DEGs. Hub genes were screened using LASSO analysis and SVM-RFE analysis. To further explore the differences of the expression of hub genes in healthy control and RA patient synovial tissues, we calculated the ROC curves and AUC. The expression levels of hub genes were verified in synovial tissues of normal and RA rats by qRT-PCR and western blot. Furthermore, the CIBERSORTx was implemented to assess the differences of infiltration in 22 immune cells between normal and RA synovial tissues. We explored the association between hub genes and infiltrating immune cells.

**Results:**

CRTAM, CXCL13, and LRRC15 were identified as RA's potential hub genes by machine learning and LASSO algorithms. In addition, we verified the expression levels of three hub genes in the synovial tissue of normal and RA rats by PCR and western blot. Moreover, immune cell infiltration analysis showed that plasma cells, T follicular helper cells, M0 macrophages, M1 macrophages, and gamma delta T cells may be engaged in the development and progression of RA.

**Conclusions:**

In brief, our study identified and validated that three hub genes CRTAM, CXCL13, and LRRC15 might involve in the pathological development of RA, which could provide novel perspectives for the diagnosis and treatment with RA.

## 1. Introduction

Rheumatoid arthritis (RA) is recognized as a chronic inflammatory disease featured by pathological synovial inflammation, affecting roughly 1% of the world population [1]. Irreversible synovial inflammation and joint destruction pose significant adverse effects on the quality of life for RA patients. Currently, non-steroidal anti-inflammatory drugs and disease-modifying anti-rheumatic drugs are the primary clinical agents for the therapy of RA [2]. However, these treatments are usually associated with adverse reactions, including increased risks of infection and tumor prevalence [3]. Early diagnosis and treatment of RA can significantly delay the development of the disease and enhance the quality of life of patients [4]. Currently, the underlying pathogenesis of RA is not fully elucidated. Many studies showed that identifying disease-related hub genes could better understand the pathogenesis of disease [5, 6]. Therefore, the identification of novel hub genes in the synovial tissue will unravel the underlying mechanisms of RA and facilitate the development of effective treatment strategies.

In recent years, many studies have found that immune cell infiltration plays a pivotal role in the development and progression of RA. Key immune cells, such as macrophages, plasma cells, T cells, and natural killer cells, are involved in the pathogenesis of RA [7]. These cells secrete numerous inflammatory mediators, including cytokines, chemokines, and matrix-degradative enzymes, leading to joint inflammation and bone destruction. A study has indicated that plasma cells play a vital role in the pathogenesis of RA [8]. Plasma cells, also known as effector B cells, play a role in the humoral immune response by producing large amounts of cytokines in the immune system, including IL-17 and TNF-*α* [9]. A large number of activated T cells invade the synovial tissue and release many inflammatory cytokines, causing inflammation in the synovial membrane of RA and ongoing tissue damage [10]. In addition, macrophages contributed to RA synovial inflammation via the generation of pro-inflammatory mediators and further promoted the pathophysiology of RA [11]. Nevertheless, the immune modulatory mechanisms of RA in synovial tissue lacked in-depth study. Moreover, investigating the association between immune cells and hub genes could better elucidate the pathogenesis of RA [12]. Consequently, the role of immune cells in RA needs to be further evaluated and the relationship between immune cells and hub genes needs to be comprehensively explored.

Despite a great deal of research confirming the aberrant expression of immune cells in RA, the role of the immune system in RA is not yet fully elucidated [13]. Evidence has demonstrated that imbalances in the immune system could lead to synovial inflammation and the formation of synovial vascular hyperplasia [14]. RA is an autoimmune disease, yet it has a long course and poor prognosis, eventually leading to joint deformity and even disability. Therefore, exploring immune dysfunction in RA may help to provide relevant insights into the etiopathogenesis of RA and contribute to the development of new therapeutic strategies. Recently, bioinformatics analysis of microarray data has contributed to the understanding of the molecular mechanisms of RA occurrence and development and identified potential hub genes that might be potentially used for clinical diagnosis of RA [15–17]. A study found that RPS29 and RPL10A were highly expressed in the synovial tissues of RA patients by bioinformatics analysis [18]. Based on the WGCNA, FADD, CXCL2, and CXCL8 may be regarded as potential diagnostic biomarkers for RA [19]. By bioinformatics analysis, FN1, VEGFA, HGF, SERPINA1, MMP9, PPBP, CD44, FPR2, IGF1, and ITGAM were recognized as the hub genes related to synovial macrophages in RA [20]. Furthermore, TNFAIP6/TSG6 and HSP90AB1/HSP90 were identified as new biomarkers for RA by cross-tissue transcriptomic analysis leveraging machine learning approaches [21]. However, there are some shortcomings in these findings. For example, the sample sizes of some findings are too small and the screening criteria too low to be representative. Besides, some of the findings were not experimentally validated. Therefore, there is an urgent need for more in-depth analysis and experimental validation of potential biomarkers of RA for a comprehensive study.

In the study, we attempted to identify vital molecular factors to illustrate the underlying mechanisms of RA and offer potential targets for the treatment of RA. Firstly, we obtained RA datasets from the Gene Expression Omnibus (GEO) database and recognized differentially expressed genes (DEGs) by differential expression analysis (GSE77298 and GSE55457). Secondly, enrichment analysis was applied to these DEGs, including Gene Ontology (GO), Kyoto Encyclopedia of Genes and Genomes (KEGG), and Gene Set Enrichment Analysis (GSEA). Then, integrated bioinformatics analysis and machine learning strategies were employed to identify hub genes for validation using other datasets (GSE55235 and GSE12021). Finally, we estimated the immune profile of RA and the relationship between immune cells and hub genes. To further verify the above analysis results, we detected the mRNA and protein expression levels of the hub genes in the synovial tissues of RA rats. Collectively, these findings may provide novel insights into the molecular immune mechanisms and reveal potential novel approaches to the treatment of RA.

## 2. Materials and Methods

### 2.1. Data Collection

Gene expression datasets of RA were searched and obtained from the public repository databases GEO (http://www.ncbi.nlm.nih.gov/geo/) using the following keywords: “Rheumatoid Arthritis” (keyword), “Homo sapiens” (organism), “synovial tissue” (attribute name), and “sample count” >20. The GSE77298 from GPL570 platform was produced and included data of synovial tissues from 16 RA and 7 healthy control samples [22]. The microarray dataset GSE55457 from the GPL96 platform contained 13 samples of RA synovial tissue and 10 samples of healthy control synovial tissue [23]. The GSE55235 included 10 RA synovial samples and 10 normal synovial samples on the basis of the GPL96 platform [23]. The GSE12021 from the GPL96 platform included 9 normal synovial samples and 12 RA synovial samples [24]. Clinical information for the four datasets is presented in Supplementary Table 1.

### 2.2. Data Preprocessing and Differential Gene Expression Analysis

Data statistical analyses were conducted using R software v4.0.3 (http://www.r-project.org/) and Bioconductor (http://bioconductor.org/). The microarray data GSE77298 and GSE55457 were processed and normalized with the “limma” and “sva” packages of R software [25, 26], and batch correction was conducted using the Combat function from the “sva” package. Then, we utilized the R package “limma” to identify DEGs based on the datasets (GSE77298 and GSE55457), and statistical significance was set at log2FC > 2 and adjusted *p* value < 0.05. Differential expression of DEGs was visualized with the R packages “ggplot2” [27] and “pheatmap” (version 1.0.8).

### 2.3. Functional Enrichment Analysis

To explore the potential functional and molecular pathways of DEGs, we performed GO, KEGG enrichment analyses of DEGs by the “clusterProfiler” package in R [28]. *p* < 0.05 was considered to show the statistical significance. The GSEA was employed to identify the most significant functional terms between the RA and control groups. And the “c2.cp.kegg.v7.4.symbols.gmt” from the Molecular Signatures Database (MSigDB) (https://www.gsea-msigdb.org/gsea/index.jsp) was used as the reference gene set. The significant differences were identified with the *p*-value adjusted < 0.05.

### 2.4. Identification and Verification of Hub Genes

We further screened signature DEGs associated with RA using least absolute shrinkage and selection operator (LASSO) logistic regression [29] and support vector machine recursive feature elimination (SVM-RFE) [30] algorithms by five-fold cross-validation, respectively. The LASSO logistic regression was carried out the “glmnet” package in R [31]. The SVM-RFE algorithm was applied with the “e1071” R package [32]. The DEGs screened by the two algorithms were overlapped to obtain three genes as hub genes with the “venn” R package (version 1.7). The GSE55235 and GSE12021 datasets were used as external datasets to validate the hub genes screened by using the Wilcoxon rank test analysis. The receiver operating characteristic (ROC) curves and area under the curve (AUC) were also computed to evaluate the predictive effectiveness of the algorithm using the R package “pROC” [33]. And a two-sided *p* < 0.05 showed a statistically significant difference.

### 2.5. Animal Experimental Design

Sixteen Sprague-Dawley (160-180 g) rats were adaptive feeding for one week, half male and half female. All rats were purchased from the Experimental Animal Center of Southwest Medical University (Luzhou, China). First, 16 rats were randomly divided into two groups (*n* = 8 in each group): the control group and the model group. Then, the model group rats were intradermally injected with 0.15 ml of Freund's Complete Adjuvant (FCA, Sigma) at the right hind footpad to establish an adjuvanticity arthritis (AA) model [34], and the same volume of 0.9% saline was administered to the control group rats. Significant swelling of the foot pad in the model group indicated successful modeling compared to the control group. Rats were sacrificed on day 8 of the experiment. The synovial tissues of the rat were resected and placed in liquid nitrogen for further analysis, and rats were euthanized via cervical dislocation. All animal experimental procedures were approved by the Institutional Ethics Committee of South Western Medical University.

### 2.6. Evaluation of Foot Volume and Arthritis Score

Before each rat was injected at the right hind footpad (day 0), we measured the foot volume of each rat by a self-made foot volume measurement device [35]. After the 7th day of FCA injection, the right hind footpad volume of each rat was again measured. On days 0, 3, and 7 of the experiment, all rats were measured by arthritis score. The arthritis score was used to evaluate the inflammation of joints in each group of rats. The arthritis score was as follows: 0 points = normal; 1 point = mild redness or swelling of only regional parts or toes; 2 points = moderate or mild swelling of feet, pads, or ankles; 3 points = severe swelling of ankle joints or complete swelling below ankle joints; and 4 points = severely and highly swollen feet, toes, and joints, without stiffness or deformity [36].

### 2.7. Quantitative PCR Analysis

The total RNA was extracted from synovial tissue using RNAiso Plus (Takara, China) according to the manufacturer's protocol. Afterward, first-strand cDNA was synthesized from total RNA using transcriptor first-strand cDNA synthesis kit (Roche, Germany). The relative mRNA levels of CRTAM, CXCL13, and LRRC15 were measured with Stormstar SybrGreen qPCR Master Mix (DBI, Germany). Besides, *β*-actin was used as a reference gene for normalization of different transcript values. According to gene sequences in the NCBI database, specific primers were designed by the Primer Premier 5.0 software (Premier Biosoft, USA). The sequences of the PCR primers are as shown in Table 1. The relative mRNA expression levels were evaluated with the 2^-*ΔΔ*^ct method. All PCR assays were performed in triplicate.

### 2.8. Western Blot Analysis

Western blot was performed as previously described [37, 38]. Antibodies used in the study are as follows: anti-CRTAM (1 : 1000; clone EPR23786-12, AB272723, Abcam, UK), anti-LRRC15 (1 : 1000; clone EPR8188, AB150376, Abcam, UK), and anti-GAPDH (1 : 1000; clone 14C10, #2118, Cell Signaling Technology, China). The expressions of CRTAM and LRRC15 protein were analyzed by ImageJ software (http://rsb.info.nih.gov/ij/index.html).

### 2.9. Correlation Analysis between Immune Cells and Hub Genes

To further study the correlation of immune cell and hub genes in RA, we used the CIBERSORTx (https://cibersort.stanford.edu) to assess the differences of infiltration of 22 immune cells between normal and RA synovial tissues. Then, the relationship between the 22 immune cells was revealed using the “corrplot” package of R software. Additionally, we also conducted a correlation analysis of 22 immune cells with hub genes by Spearman's rank correlation analysis. All analysis results were visualized using “ggplot2” package, and *p* < 0.05 was considered statistically significant.

### 2.10. Statistical Analysis

All statistical analyses were performed using the R version 4.0.3, SPSS 17.0, and GraphPad Prism 8. The Wilcox test was used to compare the intergroup difference between two groups. A *p*-value < 0.05 was considered statistically significant.

## 3. Results

### 3.1. Identification of Differentially Expressed Genes

**Figure 1:**
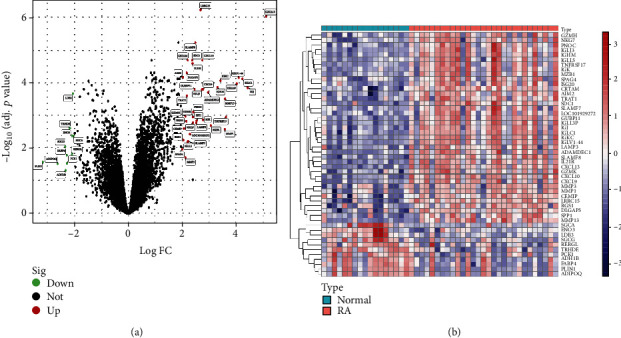
DEGs analysis in normal and rheumatoid arthritis cases. (a) The volcano plots for DEGs. There are 36 upregulated DEGs and 8 downregulated DEGs. (b) The heat map showing profiles of the DEGs.

To study gene expression in RA, we integrated the two microarray datasets GSE77298 and GSE55457 into a complete dataset using the “sva” R package. And differential expression analysis showed a total of 44 DEGs in the combined dataset, of which 36 DEGs were upregulated and 8 DEGs were downregulated (Figure 1 and Supplementary Table 2).

### 3.2. Function and Pathway Enrichment

**Figure 2:**
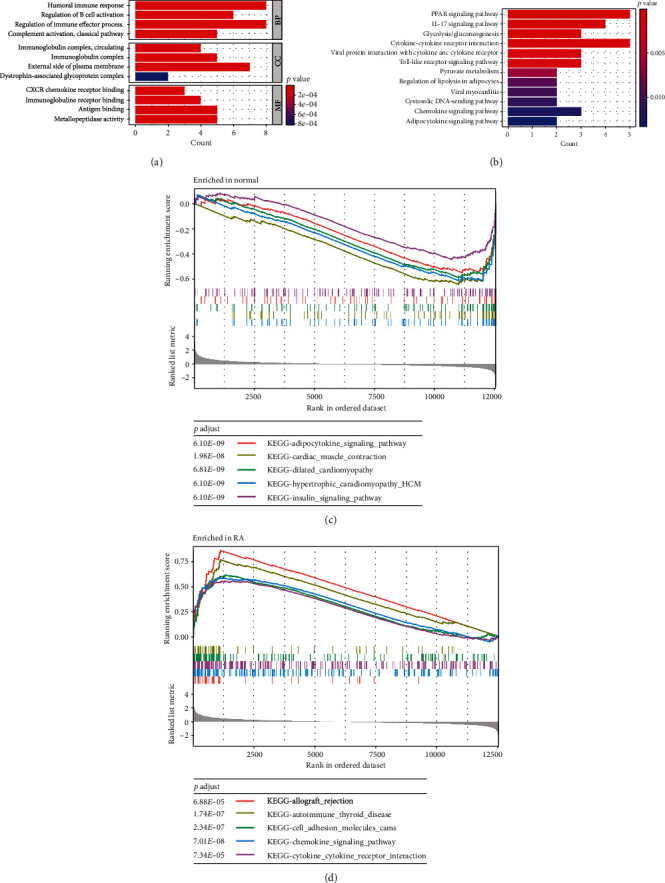
The functional enrichment analyses of DEGs. (a) The GO analyses results. (b) The KEGG analysis results. (c) The GSEA analysis revealing the five remarkable signal pathways in normal group. (d) The GSEA analysis depicting the five significant signal pathways in RA group.

In the GO enrichment analysis of DEGs (Figure 2(a) and Supplementary Table 3), biological processes (BP) terms were related to humoral immune response and regulation of immune effector process; cellular components (CC) terms were correlated with the external side of the plasma membrane and inflammasome complex; and molecular functions (MF) terms were associated with antigen binding and immunoglobulin receptor binding. KEGG enrichment analysis showed that DEGs were significantly enriched in PPAR signaling pathway, cytokine-cytokine receptor interaction, and IL-17 signaling pathway (Figure 2(b) and Supplementary Table 4). To further clarify the differences in functional and biological pathways between RA patients and healthy controls, we performed GSEA analysis of the DEGs and screened significant enriched signaling pathways. As shown in Figures 2(c) and 2(d), and Supplementary Table 5, cell adhesion molecules, chemokine signaling pathway, cytokine-cytokine receptor interaction, and autoimmune thyroid disease were enriched in the RA group.

### 3.3. Identification of Hub Genes

**Figure 3:**
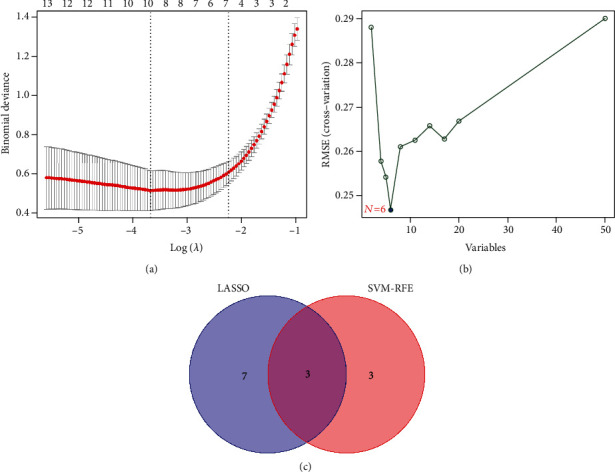
Identification of hub genes related to RA. (a) The hub genes identified by the LASSO analysis. (b) The hub genes screened using SVM-RFE algorithm. (c) Venn diagram showing the intersection among hub genes of both algorithms.

After identifying DEGs, we adopted LASSO regression analysis and SVM-RFE analysis for feature selection. Firstly, we performed the LASSO analysis to identify 10 key genes (LRRC15, CXCL13, CRTAM, DLGAP5, CXCL9, LDB3, PNOC, TNFRSF17, SGCA, and ADH1B) from 44 DEGs (Figure 3(a)). Then, six signature genes, including SDC1, LRRC15, CXCL13, CRTAM, IGLJ3, and AIM2, were identified from 44 DEGs by the SVM-RFE analysis (Figure 3(b)). By combining LASSO regression analyses and SVM-RFE analyses, three hub genes related to RA were screened, namely, LRRC15, CXCL13, and CRTAM (Figure 3(c)).

### 3.4. Validation of Hub Genes

**Figure 4:**
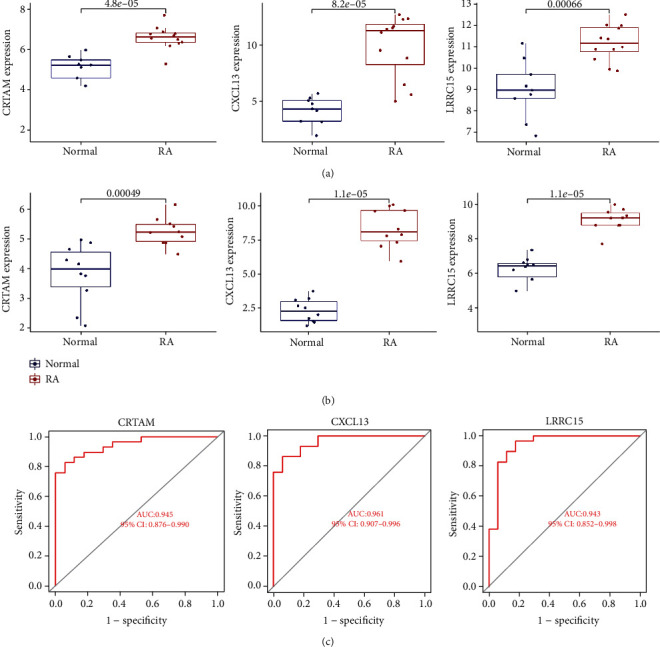
Validation of the hub genes. (a, b) Expression levels of CRTAM, CXCL13, and LRRC15 between normal and RA synovial tissues. Verification using two external datasets (GSE12021 and GSE55235). (c) The ROC curves analysis.

In order to ensure the significance and accuracy of the results, two external datasets (GSE12021 and GSE55235) were used for external validation to validate three hub genes. The results displayed that CRTAM, CXCL13, and LRRC15 were all significantly upregulated in RA tissues compared to normal tissues (*p* < 0.01) (Figures 4(a) and 4(b)). In general, the findings confirm the reliability of the above results. To evaluate the discriminative power of hub genes for the purpose of RA tissue and normal tissue, we performed ROC analysis on the two external datasets. According to the ROC analysis results, three hub genes had high performance in distinguishing between RA and normal tissue, suggesting that they could serve as potential early diagnostic biomarkers. The ROC curve analysis showed that the AUC values of CRTAM, CXCL13, and LRRC15 were 0.945, 0.961, and 0.943, respectively, suggesting that the three biological markers had a high accuracy of predictive value (Figure 4(c)).

### 3.5. Effect of FCA on Foot Volume and Arthritis Score Variations in Rats

At day 0, there was no significant difference in foot volume and arthritis score between the control group and the model group (*p* > 0.05) (Supplementary Figure 1). Compared to the control group, the model group of right foot volumes and arthritis scores were significantly increased (*p* < 0.01) at day 7, which indicated that building the AA rat model was successful.

### 3.6. qRT-PCR Validation of Hub Genes

**Figure 5:**
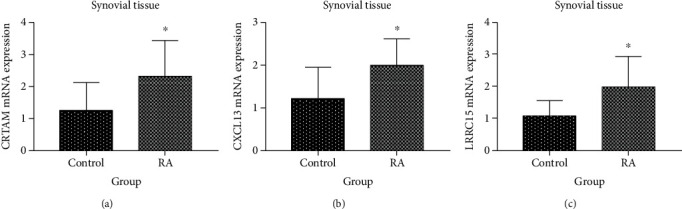
The mRNA expressions of hub genes were validated by qRT-PCR. ^∗^*p* < 0.05. (a–c) The mRNA expressions of CRTAM, CXCL13, and LRRC15 between control and RA rat synovial tissues (*n* = 8).

We examined the relative mRNA expression of CRTAM, CXCL13, and LRRC15 in rats' synovial tissue by qRT-PCR (Figure 5). Compared to the control group, the mRNA expression of CRTAM, CXCL13, and LRRC15 was remarkably higher in the model group (*p* < 0.05).

### 3.7. Western Blot Validation of Hub Genes

**Figure 6:**
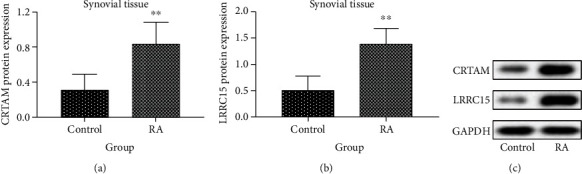
The protein expressions of hub genes were validated by western blot. ^∗∗^*p* < 0.001. (a–c) The protein expressions of CRTAM and LRRC15 between control and RA rat synovial tissues (*n* = 8).

We tested the protein expression of CRTAM and LRRC15 in synovial tissues of rats by western blot. As shown in Figure 6, the protein expression of CRTAM and LRRC15 was remarkably increased in the model group compared to the control group (*p* < 0.05).

### 3.8. Infiltration of Immune Cells

**Figure 7:**
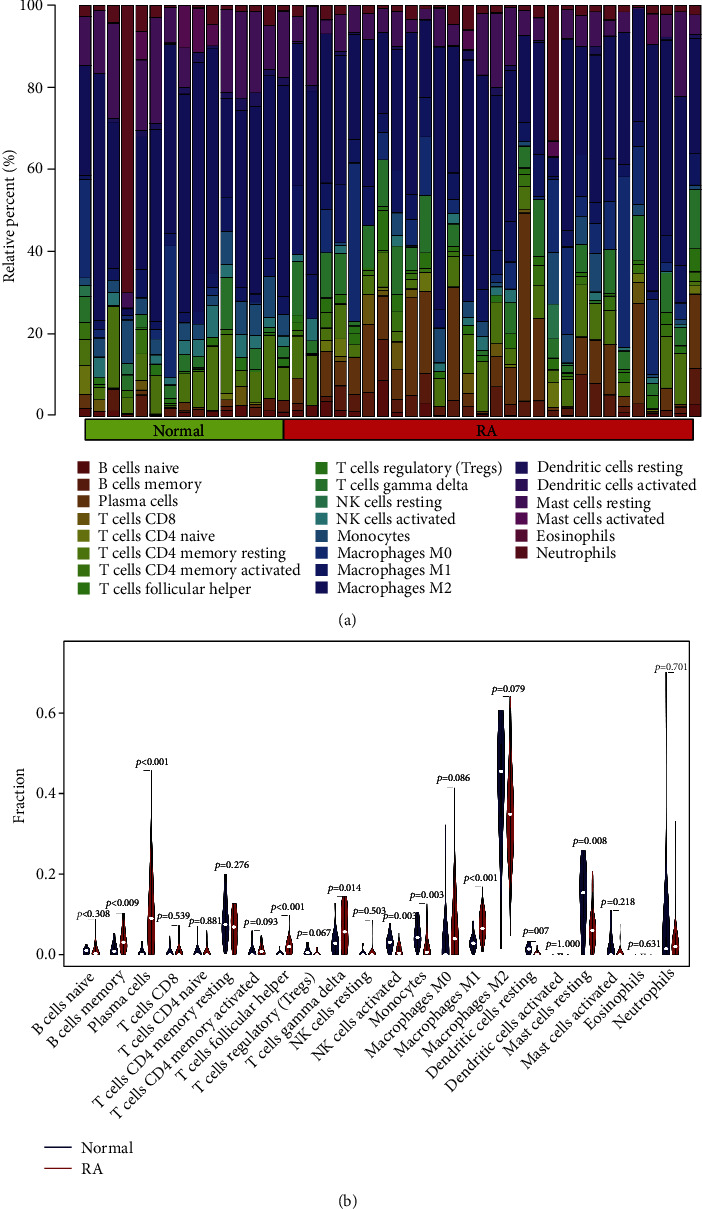
Assessment of immune cell infiltration. (a) Immune cell types and proportions between normal controls and RA patients. (b) Violin diagram of the level of 22 immune cells' infiltration in the normal and RA synovial samples.

**Figure 8:**
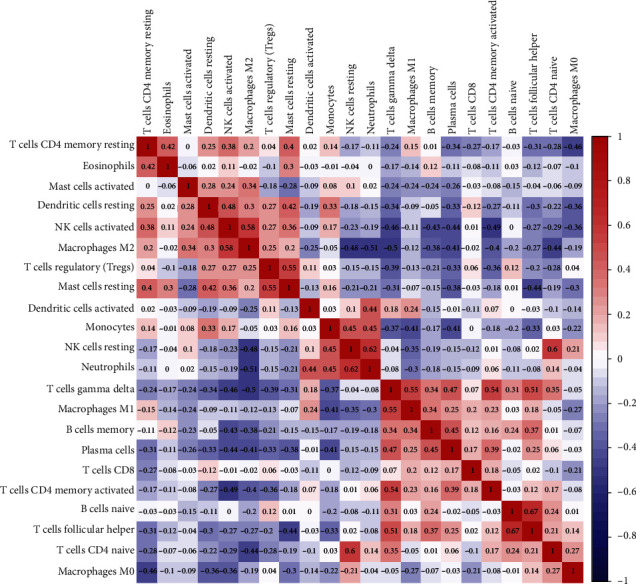
Correlation heat map of 22 types of immune cells.

**Figure 9:**
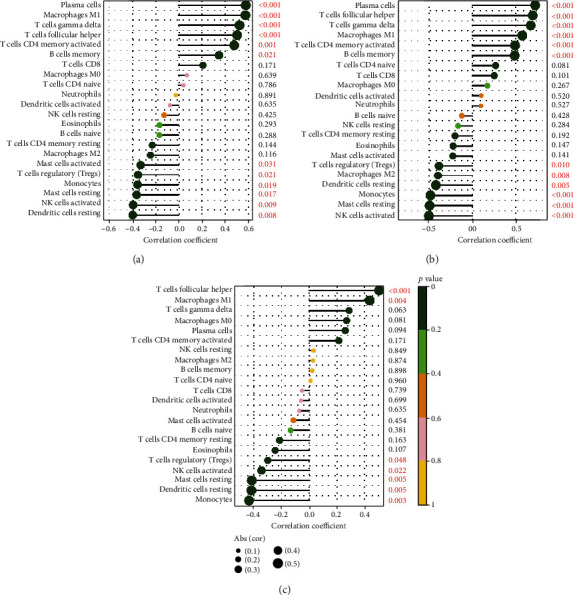
Correlation between hub genes and infiltrating immune cells. (a–c) Association of CRTAM, CXCL13, and LRRC15 with 22 immune cells.

Furthermore, we also used the CIBERSORTx algorithm to assess the levels of 22 immune cells' infiltration (Figure 7). The results showed that plasma cells (*p* < 0.001), T follicular helper cells (*p* < 0.001), M1 macrophages (*p* < 0.001), B cells memory (*p* < 0.05), gamma delta T cells (*p* < 0.05), and monocytes (*p* < 0.05) had significantly higher expression in RA synovial tissues compared to normal synovial tissues. As shown in Figure 8, neutrophils and resting NK cells (0.62), follicular helper T cells, and naive B cells (0.67) displayed significant positive correlations, respectively. Neutrophils and M2 macrophages (-0.51), gamma delta T cells, and M2 macrophages (-0.5) displayed a significant negative correlation, respectively. Since these three genes were significantly involved in immune-related pathways, we further evaluated their association with immune cells. Results showed that CRTAM displayed a positive correlation with plasma cells (*r* = 0.578, *p* < 0.001), and M1 macrophages (*r* = 0.575, *p* < 0.001) showed a negative correlation with resting dendritic cells (*r* = -0.393, *p* < 0.01) and activated NK cells (*r* = -0.390, *p* < 0.01) (Figure 9(a)). Besides, CXCL13 showed a positive correlation with plasma cells (*r* = 0.719, *p* < 0.001) and follicular helper T cells (*r* = 0.691, *p* < 0.001) and a negative correlation with activated NK cells (*r* = -0.500, *p* < 0.001) and resting mast cells (*r* = -0.491, *p* < 0.001) (Figure 9(b)). Furthermore, LRRC15 showed a positive correlation with follicular helper T cells (*r* = 0.499, *p* < 0.001) and M1 macrophages (*r* = 0.431, *p* < 0.01) and a negative correlation with monocytes (*r* = -0.433, *p* < 0.01) and resting dendritic cells (*r* = -0.417, *p* < 0.01) (Figure 9(c)).

## 4. Discussion

RA is pathologically manifested as chronic inflammatory cell infiltration and pannus formation in synovial tissues, leading to the destruction of articular cartilage and bone. Earlier studies showed that chronic inflammatory cell infiltration significantly affects the pathological processes of RA [39]. Immune cells are involved in mediating the inflammatory response in RA, and abnormally active immune cells may lead to excessive secretion of inflammatory cytokines, eventually exacerbating the course of RA [40, 41]. Therefore, the identification of hub genes and genetic signatures, and analyzing the characterization of RA immune cell infiltration are essential to assess the efficacy of various therapeutic approaches for RA patients. Through the above analysis, we screened three hub genes, namely, CRTAM, CXCL13, and LRRC15, and performed further validation by qPCR and western blot. In addition, the association between hub genes and immune cell infiltration was further analyzed by the CIBERSORTx algorithm. Collectively, we identified the hub genes that are associated with RA by machine learning and LASSO algorithms and investigated the role of immune cell infiltration in RA, which could offer a basis for the diagnosis and treatment of RA.

In this study, we screened four datasets from the GEO database associated with RA and normal synovial tissue. With R software, a total of 44 DEGs were identified between RA and normal synovial tissue, including 36 upregulated and 8 downregulated DEGs, respectively. GO analysis revealed that hub genes were involved in regulating immune-related signaling pathways, including humoral immune responses, cytokine activity, and cytokine-mediated signaling pathway. KEGG analysis revealed that the PPAR signaling pathway, cytokine-cytokine receptor interaction, and IL−17 signaling pathway were related to DEGs. GO and KEGG enrichment studies indicated that immune responses and immune cytokines were involved in the pathological development of RA, leading to an inflammatory response in the synovial of RA, which induced joint pain and swelling. Next, we conducted GSEA analysis to seek out potential pathways associated with RA. Besides, three hub genes (CRTAM, CXCL13, and LRRC15) were selected by LASSO regression analysis and SVM-RFE analysis. To further validate the relationship between hub genes and RA, 3 hub genes were analyzed via PCR and western blot. We found that the mRNA expression levels of CRTAM, CXCL13, and LRRC15 were highly expressed in the synovial tissue of RA rats, and the protein expression of CRTAM and LRRC15 was highly expressed in the synovial tissue of RA rats, which was consistent with our previous screening results.

The class-I MHC-restricted T cell-associated molecule (CRTAM) is an immunoglobulin that was initially detected to be highly expressed in activated CD8^+^ T cells and NKT cells [42]. CRTAM might promote IFN-*γ* secretion, which contributes to an antitumor effect [43]. Early studies demonstrated that CRTAM-deficient mice could result in reduced protective immunity against viral infections in vivo [44]. The discovery of CRTAM^+^ NK cells in the bone marrow of patients with acute lymphoblastic leukemia may have potential immune response suppressive properties [45]. Many studies have confirmed that CRTAM is closely associated with the inflammatory response. CRTAM was found to enhance the degree of inflammation in intestinal infections, which suggested that CRTAM could be involved in the inflammatory immune response [46]. Furthermore, the crtam^−/−^ mice infected with salmonella exhibit reduced Th17 responses and thereby decreased levels of inflammation, suggesting that CRTAM may be one of the important contributors to intestinal inflammation [47].

The C-X-C motif ligand 13 (CXCL13) is a key chemo-tactic cytokine that promotes the migration and aggregation of B lymphocytes and is widely involved in various immune responses [48]. A study suggested that the occurrence of RA may be related to CXCL13 overexpression [49]. Clinical studies confirmed that the serum expression level of CXCL13 in RA patients was significantly higher than in healthy controls [50]. Positive expression of CXCL13 was closely correlated with the clinical severity of RA, suggesting that it may be a predictive biomarker for RA treatment [51]. Moreover, the synovial fluid levels of CXCL13 were significantly higher in RA patients than in healthy individuals [52]. Furthermore, the expression level of CXCL13 was significantly increased in the synovial tissue of CIA mice compared to normal tissues, which was consistent with our findings [53].

The leucine-rich repeat containing 15 (LRRC15) is a leucine-rich transmembrane protein, that belongs to the leucine-rich repeat superfamily. LRRC15 was involved in cell-cell and cell-extracellular matrix interactions and performed a key function in signal transduction [54]. Recent studies identified that LRRC15 was an important factor contributing to cartilage damage in osteoarthritis [55]. In addition, LRRC15 protein was found in the articular cartilage of OA patients by immunostaining [55]. Previous studies have also demonstrated that LRRC15 may play a vital role in reducing bone resorption and promoting bone formation by acting as a suppressor of NF-*κ*B [56]. Furthermore, LRRC15 was found to be overexpressed in aggressive cancer cells, such as breast cancer [57], ovarian cancer [58], and osteosarcoma [59].

To further explore the correlation between hub genes and immunity, we also assessed 22 immune cell infiltration levels in RA and normal samples. We found that plasma cells, T follicular helper cells, M0 macrophages, M1 macrophages, gamma delta T cells, T regulatory cells, NK cells, monocytes, resting dendritic cells, and resting mast cells were significantly different between normal and RA synovial tissues. Through the secretion of inflammatory cytokines, the inflammatory cells in synovial tissue play a vital role in the pathogenesis of RA. It has been found that in RA synovial tissue, a variety of inflammatory cells are recruited and activated, including innate immune cells (plasma cells, macrophages, dendritic cells, and NK cells) and adaptive immune cells (T and B cells), which contribute to inflammation and damage in RA joints [60]. Previous studies have revealed that immune cell infiltration was observed in the synovial tissue of RA patients, including T cells, plasma cells, dendritic cells, and macrophages, which was consistent with our results [61]. Plasma cells could produce autoantibodies upon activation and play a key role in the immune response to RA [62]. Interestingly, a study reported found that the number of plasma cells was significantly increased in the bone marrow of RA mice [63]. T follicular helper cells were a separate subset of CD4^+^ T cells and were essential for B-cell-mediated immunity [64]. A study confirmed the presence of large numbers of T follicular helper cells in the synovial tissue of RA patients [65]. Macrophages are central to the pathophysiology of RA. Macrophages are conventionally classified as pro-inflammatory (M1) and anti-inflammatory (M2) functional types. They are a major source of pro-inflammatory cytokines and chemokines, which promote local tissue destruction and inflammatory responses in RA by activating large numbers of immune and non-immune cells and secreting various cytokines [66]. Dendritic cells play a crucial role in the pathological development of RA as initiators and modulators of adaptive immune responses [67].

CRTAM suggested a positive association with plasma cells, and T gamma delta cells and M1 macrophages under the activation and negative associations with resting dendritic cells. CXCL13 showed positive associations to plasma cells and T follicular helper cells and showed negative associations to resting mast cells and resting dendritic cells. LRRC15 showed positive associations to T follicular helper cells and a negative association with resting dendritic cells and resting mast cell. It has been reported that CXCL13 was secreted by T helper follicular cells, which recruit B cells to the germinal center (GCs) and participate in the immune response [68]. In another study, it was reported that CXCL13 is also produced by the peripheral helper T cells in the RA joint [69]. LRRC15 belongs to the leucine-rich repeat superfamily, and numerous studies have shown that LRRC15 is induced and highly expressed in various tumor types [58, 70]. Previous studies have demonstrated that LRRC15 was found to have upregulated expression in RA osteoblasts [71]. Due to the lack of research on RA-related hub genes, further exploration of RA-related hub genes and immune cell interactions is needed based on the above screening results.

However, there are still a few limitations to our study. Firstly, the valid sample size of the dataset in the GEO database is too small, which leads to some bias in the bioinformatics analysis. Additional samples need to be collected to further assess the reliability of our predictive hub genes. Because of the difficulty of collecting human synovial tissue, we used synovial tissue from the classical AA rat model for experimental validation. In the next work, we will try to gather human synovial tissue samples to further study the expression of hub genes in healthy subjects and RA patients. Finally, the clinical value of our findings needs to be validated in further in vitro experiments and clinical trials.

## 5. Conclusion

In conclusion, we identified and validated three hub genes (CRTAM, CXCL13, and LRRC15) in RA synovial tissue. Besides, the relationship between hub genes and immune cells was assessed to investigate the molecular mechanism and biological functions of RA. The findings may provide novel targets for the diagnosis and treatment of RA.

## Figures and Tables

**Table 1:** Primer sequences of hub genes.

Gene	Forward	Reverse
CRTAM	5′-GACGCCTTTCCAGCCAACT-3′	5′-GTGGAACACTTCAGCACAACAG-3′
CXCL13	5′-CAGCCCTGCTTCTTCTACTGG-3′	5′-GCTCACCTTGGAACACCTACAT-3′
LRRC15	5′-GACGCCTTTCCAGCCAACT-3′	5′-GTGGAACACTTCAGCACAACAG-3′
*β*-Actin	5′-CAGGTCATCACTATCGGCAAT- 3′	5′-CTTTACGGATGTCAACGTCACAC-3′

## Data Availability

All data generated or analyzed during this study are included in this published article. Requests for material should be made to the corresponding authors.

## Supplementary Materials

Supplementary Materials Table S1 The clinical characteristic of four datasets. Table S2. A total of 44 DEGs in the combined dataset, of which 36 DEGs were upregulated and 8 DEGs were downregulated. Table S3. The GO enrichment analysis. Table S4. The KEGG enrichment analysis. Table S5. The GSEA enrichment analysis. Figure S1. The arthritis profile of AA rat. (a) The right foot swelling variation of rats. (b) The arthritis scores variation of rats. (*Supplementary Materials*).
