# Supplementary material for: Identification and Validation of Hub Genes for Predicting Treatment Targets and Immune Landscape in Rheumatoid Arthritis
Source: Biomed Res Int. 2022 Oct 22;2022:8023779. doi: 10.1155/2022/8023779 (PMC9617710; doi:10.1155/2022/8023779)
Supplement: Supplementary Materials — Table S1 The clinical characteristic of four datasets. Table S2. A total of 44 DEGs in the combined dataset, of which 36 DEGs were upregulated and 8 DEGs were downregulated. Table S3. The GO enrichment analysis. Table S4. The KEGG enrichment analysis. Table S5. The GSEA enrichment analysis. Figure S1. The arthritis profile of AA rat. (a) The right foot swelling variation of rats. (b) The arthritis scores variation of rats. (Supplementary Materials). [file 8023779.f1.docx]

**Supplementary Table 1 |** The detail information on the gene expression profiles of patients with rheumatoid arthritis (RA).

| Datasets | Normal | | | | Rheumatoid arthritis | | | | Platform |
| --- | --- | --- | --- | --- | --- | --- | --- | --- | --- |
|  | Female | Male | Total | Age(years) | Female | Male | Total | Age(years) |  |
| GSE77298 | NA | NA | 7 | NA | NA | NA | 16 | NA | GPL570 |
| GSE12021 | 3 | 6 | 9 | 50.2±21.9 | 9 | 3 | 12 | 64.8±10.4 | GPL96 |
| GSE55235 | NA | NA | 10 | NA | NA | NA | 10 | NA | GPL96 |
| GSE55457 | 2 | 8 | 10 | 51.0±19.7 | 10 | 3 | 13 | 64.6±9.9 | GPL96 |

**Supplementary Table 2 |** A total of 44 differentially expressed genes (DEGs) in the combined dataset, of which 36 DEGs were upregulated and 8 DEGs were downregulated.

| id | logFC | AveExpr | P.Value | adj.P.Val |
| --- | --- | --- | --- | --- |
| PLIN1 | -3.097308626 | 6.884970746 | 0.002322226 | 0.026905229 |
| ADIPOQ | -2.514596009 | 7.837572829 | 0.002898754 | 0.0306213 |
| PCK1 | -2.319612843 | 6.07201937 | 0.00268833 | 0.029278701 |
| ADH1B | -2.287934962 | 6.93502211 | 0.005745423 | 0.048415226 |
| FABP4 | -2.16432231 | 9.782623458 | 0.001119488 | 0.016790985 |
| SGCG | -2.12208615 | 4.477570459 | 0.000434925 | 0.009077402 |
| TRHDE | -2.105316761 | 5.903783238 | 4.99E-05 | 0.0024368 |
| RERGL | -2.049847053 | 5.142778437 | 0.000995658 | 0.015586216 |
| ISG20 | 2.010540643 | 6.697115278 | 2.20E-05 | 0.001491911 |
| PLA2G2D | 2.024142608 | 4.664348646 | 0.00010094 | 0.003663203 |
| GZMH | 2.041565699 | 5.766084412 | 1.12E-05 | 0.001030004 |
| AIM2 | 2.057469486 | 7.935221028 | 8.07E-08 | 6.75E-05 |
| DLGAP5 | 2.067888787 | 5.771462025 | 4.57E-08 | 5.03E-05 |
| SLAMF7 | 2.07724162 | 5.747764892 | 0.000229821 | 0.006133093 |
| CRTAM | 2.111919766 | 5.681085096 | 1.26E-08 | 1.98E-05 |
| IGLL5 | 2.189082359 | 7.990435267 | 0.000242656 | 0.006350068 |
| TRAT1 | 2.231593122 | 4.370444809 | 1.43E-06 | 0.000249593 |
| LOC101929272 | 2.248528806 | 4.957691164 | 0.000103779 | 0.003703063 |
| GUSBP11 | 2.288550243 | 7.91465862 | 5.26E-07 | 0.000153338 |
| PNOC | 2.30341787 | 5.92268964 | 7.48E-06 | 0.00077517 |
| SDC1 | 2.306688413 | 7.442173586 | 1.09E-08 | 1.98E-05 |
| SPP1 | 2.328518835 | 10.0363911 | 2.10E-05 | 0.00148035 |
| GZMK | 2.339407351 | 7.540097317 | 1.19E-05 | 0.001066257 |
| SLAMF8 | 2.366223145 | 8.029202694 | 1.44E-09 | 5.62E-06 |
| IL21R | 2.380558461 | 5.849978291 | 1.84E-08 | 2.56E-05 |
| NKG7 | 2.432439699 | 5.205657788 | 2.67E-05 | 0.001664233 |
| LAMP3 | 2.480301269 | 6.355021912 | 2.72E-05 | 0.001679122 |
| LRRC15 | 2.603684169 | 9.293272687 | 4.86E-11 | 6.10E-07 |
| IGLJ3 | 2.680592594 | 7.05233829 | 5.96E-07 | 0.000158958 |
| CXCL10 | 2.698527894 | 8.477074166 | 1.21E-08 | 1.98E-05 |
| IGK | 2.729204505 | 5.546797061 | 1.74E-05 | 0.001346671 |
| CXCL9 | 2.764187023 | 9.354300424 | 5.10E-07 | 0.000152343 |
| ADAMDEC1 | 2.95236844 | 7.398245065 | 8.36E-07 | 0.000188505 |
| MZB1 | 3.053828519 | 6.34529241 | 3.65E-05 | 0.002027764 |
| IGHM | 3.21634435 | 7.807437797 | 4.02E-07 | 0.000132789 |
| IGKC | 3.292920797 | 8.416242267 | 1.85E-07 | 8.28E-05 |
| MMP13 | 3.442384601 | 5.713180255 | 2.01E-06 | 0.000314414 |
| MMP1 | 3.45120525 | 10.09100895 | 5.81E-05 | 0.002686489 |
| TNFRSF17 | 3.587808976 | 4.73204195 | 1.20E-05 | 0.001066257 |
| IGLL3P | 3.690040933 | 9.074960568 | 2.75E-07 | 0.000101364 |
| IGLV1-44 | 3.900060143 | 8.109756335 | 9.95E-08 | 6.79E-05 |
| IGLC1 | 3.949501169 | 9.681248758 | 1.36E-07 | 7.39E-05 |
| IGJ | 4.146811491 | 7.99358653 | 4.60E-07 | 0.000141543 |
| CXCL13 | 5.12941383 | 6.505719657 | 1.49E-10 | 9.35E-07 |

**Supplementary Table 3 |** The Gene Ontology (GO) enrichment analysis of DEGs.

| ONTOLOGY | ID | Description | pvalue | Count |
| --- | --- | --- | --- | --- |
| BP | GO:0006959 | humoral immune response | 5.49E-07 | 8 |
| BP | GO:0050864 | regulation of B cell activation | 2.01E-06 | 6 |
| BP | GO:0002697 | regulation of immune effector process | 2.86E-06 | 8 |
| BP | GO:0002449 | lymphocyte mediated immunity | 6.09E-06 | 7 |
| BP | GO:0006958 | complement activation, classical pathway | 7.81E-06 | 5 |
| BP | GO:0002455 | humoral immune response mediated by circulating immunoglobulin | 1.24E-05 | 5 |
| BP | GO:0006956 | complement activation | 2.41E-05 | 5 |
| BP | GO:0006910 | phagocytosis, recognition | 2.59E-05 | 4 |
| BP | GO:0042113 | B cell activation | 3.89E-05 | 6 |
| BP | GO:0016042 | lipid catabolic process | 4.99E-05 | 6 |
| BP | GO:0042742 | defense response to bacterium | 5.41E-05 | 6 |
| BP | GO:0008037 | cell recognition | 7.53E-05 | 5 |
| BP | GO:0002460 | adaptive immune response based on somatic recombination of immune receptors built from immunoglobulin superfamily domains | 7.60E-05 | 6 |
| BP | GO:0016064 | immunoglobulin mediated immune response | 7.68E-05 | 5 |
| BP | GO:0019724 | B cell mediated immunity | 8.18E-05 | 5 |
| BP | GO:0006909 | phagocytosis | 9.06E-05 | 6 |
| BP | GO:1990266 | neutrophil migration | 9.32E-05 | 4 |
| BP | GO:0006911 | phagocytosis, engulfment | 0.000105615 | 4 |
| BP | GO:0002920 | regulation of humoral immune response | 0.000133987 | 4 |
| BP | GO:0050853 | B cell receptor signaling pathway | 0.000137885 | 4 |
| BP | GO:0099024 | plasma membrane invagination | 0.000137885 | 4 |
| BP | GO:0019730 | antimicrobial humoral response | 0.000145928 | 4 |
| BP | GO:0010324 | membrane invagination | 0.000172095 | 4 |
| BP | GO:0097530 | granulocyte migration | 0.000206731 | 4 |
| BP | GO:0050871 | positive regulation of B cell activation | 0.00021206 | 4 |
| BP | GO:0071356 | cellular response to tumor necrosis factor | 0.000269644 | 5 |
| BP | GO:0002429 | immune response-activating cell surface receptor signaling pathway | 0.000316252 | 6 |
| BP | GO:0002757 | immune response-activating signal transduction | 0.000316252 | 6 |
| BP | GO:0035690 | cellular response to drug | 0.000362052 | 3 |
| BP | GO:0034612 | response to tumor necrosis factor | 0.000379907 | 5 |
| BP | GO:0050851 | antigen receptor-mediated signaling pathway | 0.000407832 | 5 |
| BP | GO:0061844 | antimicrobial humoral immune response mediated by antimicrobial peptide | 0.000425442 | 3 |
| BP | GO:0010819 | regulation of T cell chemotaxis | 0.000499632 | 2 |
| BP | GO:0051251 | positive regulation of lymphocyte activation | 0.000625083 | 5 |
| BP | GO:0045445 | myoblast differentiation | 0.000635306 | 3 |
| BP | GO:0070098 | chemokine-mediated signaling pathway | 0.000679386 | 3 |
| BP | GO:0051591 | response to cAMP | 0.000823199 | 3 |
| BP | GO:1901739 | regulation of myoblast fusion | 0.000843414 | 2 |
| BP | GO:2000479 | regulation of cAMP-dependent protein kinase activity | 0.000843414 | 2 |
| BP | GO:1990868 | response to chemokine | 0.000901817 | 3 |
| BP | GO:1990869 | cellular response to chemokine | 0.000901817 | 3 |
| BP | GO:0097529 | myeloid leukocyte migration | 0.000908864 | 4 |
| BP | GO:0033622 | integrin activation | 0.00100523 | 2 |
| BP | GO:0030595 | leukocyte chemotaxis | 0.001070263 | 4 |
| BP | GO:0030593 | neutrophil chemotaxis | 0.001073007 | 3 |
| BP | GO:0002696 | positive regulation of leukocyte activation | 0.001113218 | 5 |
| BP | GO:0090025 | regulation of monocyte chemotaxis | 0.001180776 | 2 |
| BP | GO:1901623 | regulation of lymphocyte chemotaxis | 0.001273665 | 2 |
| BP | GO:0050867 | positive regulation of cell activation | 0.001307744 | 5 |
| BP | GO:0010818 | T cell chemotaxis | 0.001369948 | 2 |
| BP | GO:0030449 | regulation of complement activation | 0.001474051 | 3 |
| BP | GO:0072676 | lymphocyte migration | 0.001548833 | 3 |
| BP | GO:0060142 | regulation of syncytium formation by plasma membrane fusion | 0.001572642 | 2 |
| BP | GO:0022407 | regulation of cell-cell adhesion | 0.001573241 | 5 |
| BP | GO:0051607 | defense response to virus | 0.001582749 | 4 |
| BP | GO:2000406 | positive regulation of T cell migration | 0.001679027 | 2 |
| BP | GO:0002688 | regulation of leukocyte chemotaxis | 0.0018292 | 3 |
| BP | GO:0071621 | granulocyte chemotaxis | 0.001958427 | 3 |
| BP | GO:0033280 | response to vitamin D | 0.002260827 | 2 |
| BP | GO:0046683 | response to organophosphorus | 0.002281746 | 3 |
| BP | GO:1902105 | regulation of leukocyte differentiation | 0.002422582 | 4 |
| BP | GO:2000403 | positive regulation of lymphocyte migration | 0.002516584 | 2 |
| BP | GO:0002433 | immune response-regulating cell surface receptor signaling pathway involved in phagocytosis | 0.002531825 | 3 |
| BP | GO:0038096 | Fc-gamma receptor signaling pathway involved in phagocytosis | 0.002531825 | 3 |
| BP | GO:0038094 | Fc-gamma receptor signaling pathway | 0.002689704 | 3 |
| BP | GO:0007162 | negative regulation of cell adhesion | 0.002770946 | 4 |
| BP | GO:0051281 | positive regulation of release of sequestered calcium ion into cytosol | 0.002785352 | 2 |
| BP | GO:0002431 | Fc receptor mediated stimulatory signaling pathway | 0.002853551 | 3 |
| BP | GO:0051384 | response to glucocorticoid | 0.002966134 | 3 |
| BP | GO:0014074 | response to purine-containing compound | 0.003081425 | 3 |
| BP | GO:0060326 | cell chemotaxis | 0.003116271 | 4 |
| BP | GO:2000404 | regulation of T cell migration | 0.003361526 | 2 |
| BP | GO:0007520 | myoblast fusion | 0.003513546 | 2 |
| BP | GO:0030574 | collagen catabolic process | 0.003827071 | 2 |
| BP | GO:0032496 | response to lipopolysaccharide | 0.004020254 | 4 |
| BP | GO:0031960 | response to corticosteroid | 0.004034496 | 3 |
| BP | GO:0050873 | brown fat cell differentiation | 0.004320888 | 2 |
| BP | GO:0038095 | Fc-epsilon receptor signaling pathway | 0.004387755 | 3 |
| BP | GO:0071675 | regulation of mononuclear cell migration | 0.004491719 | 2 |
| BP | GO:0007584 | response to nutrient | 0.004534159 | 3 |
| BP | GO:0048545 | response to steroid hormone | 0.004556082 | 4 |
| BP | GO:0033209 | tumor necrosis factor-mediated signaling pathway | 0.004683503 | 3 |
| BP | GO:0009615 | response to virus | 0.004697222 | 4 |
| BP | GO:0045661 | regulation of myoblast differentiation | 0.004842647 | 2 |
| BP | GO:0071320 | cellular response to cAMP | 0.004842647 | 2 |
| BP | GO:0006968 | cellular defense response | 0.005022719 | 2 |
| BP | GO:0010524 | positive regulation of calcium ion transport into cytosol | 0.005022719 | 2 |
| BP | GO:0002237 | response to molecule of bacterial origin | 0.005037993 | 4 |
| BP | GO:0045619 | regulation of lymphocyte differentiation | 0.005310634 | 3 |
| BP | GO:0030098 | lymphocyte differentiation | 0.005660308 | 4 |
| BP | GO:0000768 | syncytium formation by plasma membrane fusion | 0.005773451 | 2 |
| BP | GO:0140253 | cell-cell fusion | 0.005773451 | 2 |
| BP | GO:0022408 | negative regulation of cell-cell adhesion | 0.006162708 | 3 |
| BP | GO:0006949 | syncytium formation | 0.006166915 | 2 |
| BP | GO:0042267 | natural killer cell mediated cytotoxicity | 0.00636813 | 2 |
| CC | GO:0009897 | external side of plasma membrane | 1.36E-05 | 7 |
| CC | GO:0042571 | immunoglobulin complex, circulating | 1.41E-05 | 4 |
| CC | GO:0019814 | immunoglobulin complex | 1.52E-05 | 5 |
| CC | GO:0072562 | blood microparticle | 0.002949981 | 3 |
| MF | GO:0045236 | CXCR chemokine receptor binding | 6.03E-06 | 3 |
| MF | GO:0034987 | immunoglobulin receptor binding | 1.98E-05 | 4 |
| MF | GO:0003823 | antigen binding | 2.08E-05 | 5 |
| MF | GO:0005125 | cytokine activity | 0.000102942 | 5 |
| MF | GO:0008009 | chemokine activity | 0.000130384 | 3 |
| MF | GO:0042379 | chemokine receptor binding | 0.00037627 | 3 |
| MF | GO:0048018 | receptor ligand activity | 0.000391273 | 6 |
| MF | GO:0030546 | signaling receptor activator activity | 0.000413109 | 6 |
| MF | GO:0008237 | metallopeptidase activity | 0.000551709 | 4 |
| MF | GO:0005539 | glycosaminoglycan binding | 0.001184699 | 4 |
| MF | GO:0004222 | metalloendopeptidase activity | 0.001331392 | 3 |
| MF | GO:0004175 | endopeptidase activity | 0.001793229 | 5 |
| MF | GO:0001664 | G protein-coupled receptor binding | 0.002776658 | 4 |
| MF | GO:0004252 | serine-type endopeptidase activity | 0.004737218 | 3 |
| MF | GO:0008201 | heparin binding | 0.004737218 | 3 |
| MF | GO:0050840 | extracellular matrix binding | 0.005887078 | 2 |
| MF | GO:0008236 | serine-type peptidase activity | 0.006271432 | 3 |
| MF | GO:0017171 | serine hydrolase activity | 0.006648096 | 3 |
| MF | GO:0005518 | collagen binding | 0.008763007 | 2 |
| MF | GO:0031406 | carboxylic acid binding | 0.008845814 | 3 |
| MF | GO:0043177 | organic acid binding | 0.010271706 | 3 |

**Supplementary Table 4 |** The Kyoto Encyclopedia of Genes and Genomes (KEGG) enrichment analysis of DEGs.

| ID | Description | pvalue | Count |
| --- | --- | --- | --- |
| hsa03320 | PPAR signaling pathway | 4.60E-07 | 5 |
| hsa04060 | Cytokine-cytokine receptor interaction | 0.000356294 | 5 |
| hsa04657 | IL-17 signaling pathway | 0.001083512 | 3 |
| hsa04061 | Viral protein interaction with cytokine and cytokine receptor | 0.001296218 | 3 |
| hsa04620 | Toll-like receptor signaling pathway | 0.001451695 | 3 |
| hsa00620 | Pyruvate metabolism | 0.004737633 | 2 |
| hsa04923 | Regulation of lipolysis in adipocytes | 0.006670089 | 2 |
| hsa04062 | Chemokine signaling pathway | 0.008192695 | 3 |
| hsa04623 | Cytosolic DNA-sensing pathway | 0.008381653 | 2 |
| hsa00010 | Glycolysis / Gluconeogenesis | 0.009439324 | 2 |
| hsa04920 | Adipocytokine signaling pathway | 0.009989478 | 2 |
| hsa04512 | ECM-receptor interaction | 0.015899764 | 2 |
| hsa04152 | AMPK signaling pathway | 0.02844278 | 2 |
| hsa04926 | Relaxin signaling pathway | 0.032504914 | 2 |
| hsa04371 | Apelin signaling pathway | 0.037272394 | 2 |
| hsa04936 | Alcoholic liver disease | 0.038752974 | 2 |
| hsa04964 | Proximal tubule bicarbonate reclamation | 0.049868967 | 1 |

**Supplementary Table 5 |** The Gene Set Enrichment Analysis (GSEA) enrichment analysis of DEGs.

| ID | setSize | enrichmentScore | NES | p.adjust |
| --- | --- | --- | --- | --- |
| KEGG_CHEMOKINE_SIGNALING_PATHWAY | 165 | 0.587090264 | 2.292862032 | 9.15E-09 |
| KEGG_CYTOKINE_CYTOKINE_RECEPTOR_INTERACTION | 230 | 0.556140139 | 2.262577249 | 9.15E-09 |
| KEGG_ALLOGRAFT_REJECTION | 33 | 0.835188177 | 2.466170383 | 1.15E-08 |
| KEGG_INTESTINAL_IMMUNE_NETWORK_FOR_IGA_PRODUCTION | 41 | 0.768820366 | 2.402758554 | 5.59E-08 |
| KEGG_CELL_ADHESION_MOLECULES_CAMS | 115 | 0.602680553 | 2.250229019 | 5.59E-08 |
| KEGG_LEISHMANIA_INFECTION | 67 | 0.68620873 | 2.341878426 | 9.64E-08 |
| KEGG_HYPERTROPHIC_CARDIOMYOPATHY_HCM | 75 | -0.597383751 | -2.269543137 | 1.95E-07 |
| KEGG_AUTOIMMUNE_THYROID_DISEASE | 46 | 0.741639044 | 2.382689561 | 2.30E-07 |
| KEGG_TYPE_I_DIABETES_MELLITUS | 39 | 0.757696201 | 2.333442602 | 5.92E-07 |
| KEGG_GRAFT_VERSUS_HOST_DISEASE | 35 | 0.765756599 | 2.306080583 | 6.95E-07 |
| KEGG_DILATED_CARDIOMYOPATHY | 81 | -0.572608919 | -2.218253174 | 1.62E-06 |
| KEGG_PRIMARY_IMMUNODEFICIENCY | 33 | 0.778037246 | 2.297413286 | 1.74E-06 |
| KEGG_CARDIAC_MUSCLE_CONTRACTION | 61 | -0.619321273 | -2.251090722 | 1.74E-06 |
| KEGG_NATURAL_KILLER_CELL_MEDIATED_CYTOTOXICITY | 119 | 0.555102709 | 2.083521755 | 1.75E-06 |
| KEGG_TOLL_LIKE_RECEPTOR_SIGNALING_PATHWAY | 95 | 0.597536325 | 2.164904049 | 2.68E-06 |
| KEGG_HEMATOPOIETIC_CELL_LINEAGE | 82 | 0.604911526 | 2.126955479 | 3.92E-06 |
| KEGG_ANTIGEN_PROCESSING_AND_PRESENTATION | 73 | 0.600585864 | 2.066842858 | 4.15E-05 |
| KEGG_NOD_LIKE_RECEPTOR_SIGNALING_PATHWAY | 54 | 0.651316818 | 2.155040129 | 4.15E-05 |
| KEGG_LYSOSOME | 105 | 0.534258034 | 1.966128061 | 5.09E-05 |
| KEGG_ADIPOCYTOKINE_SIGNALING_PATHWAY | 62 | -0.564553002 | -2.052250059 | 7.88E-05 |
| KEGG_FATTY_ACID_METABOLISM | 38 | -0.649540895 | -2.156976285 | 7.96E-05 |
| KEGG_T_CELL_RECEPTOR_SIGNALING_PATHWAY | 103 | 0.532534838 | 1.951359172 | 0.000162603 |
| KEGG_INSULIN_SIGNALING_PATHWAY | 127 | -0.445847003 | -1.85112367 | 0.000215364 |
| KEGG_B_CELL_RECEPTOR_SIGNALING_PATHWAY | 71 | 0.568450181 | 1.95499339 | 0.000246064 |
| KEGG_ARRHYTHMOGENIC_RIGHT_VENTRICULAR_CARDIOMYOPATHY_ARVC | 67 | -0.492249303 | -1.827812933 | 0.001166802 |
| KEGG_PPAR_SIGNALING_PATHWAY | 61 | -0.513957395 | -1.868117201 | 0.001276215 |
| KEGG_ASTHMA | 26 | 0.684838441 | 1.929698821 | 0.001872294 |
| KEGG_TIGHT_JUNCTION | 115 | -0.415419064 | -1.691222797 | 0.002053343 |
| KEGG_TYROSINE_METABOLISM | 37 | -0.587608476 | -1.938033858 | 0.002053343 |
| KEGG_FC_GAMMA_R_MEDIATED_PHAGOCYTOSIS | 87 | 0.511099309 | 1.8281958 | 0.002293765 |
| KEGG_PROXIMAL_TUBULE_BICARBONATE_RECLAMATION | 21 | -0.690950121 | -1.988078167 | 0.002334661 |
| KEGG_CELL_CYCLE | 114 | 0.464658516 | 1.733796827 | 0.003406751 |
| KEGG_P53_SIGNALING_PATHWAY | 60 | 0.533338063 | 1.782843141 | 0.005151117 |
| KEGG_ADHERENS_JUNCTION | 66 | -0.460596425 | -1.707405673 | 0.00551612 |
| KEGG_CYTOSOLIC_DNA_SENSING_PATHWAY | 46 | 0.566656569 | 1.820517276 | 0.007162334 |
| KEGG_ECM_RECEPTOR_INTERACTION | 79 | 0.483785343 | 1.691717858 | 0.008615787 |
| KEGG_CALCIUM_SIGNALING_PATHWAY | 162 | -0.346730547 | -1.486269149 | 0.015606567 |
| KEGG_NEUROACTIVE_LIGAND_RECEPTOR_INTERACTION | 240 | -0.306645607 | -1.39317956 | 0.019500581 |
| KEGG_LEUKOCYTE_TRANSENDOTHELIAL_MIGRATION | 105 | 0.425990792 | 1.567692755 | 0.019500581 |
| KEGG_SYSTEMIC_LUPUS_ERYTHEMATOSUS | 49 | 0.514166316 | 1.674548885 | 0.025882082 |
| KEGG_VALINE_LEUCINE_AND_ISOLEUCINE_DEGRADATION | 40 | -0.483609832 | -1.624414497 | 0.029373804 |
| KEGG_DRUG_METABOLISM_CYTOCHROME_P450 | 53 | -0.434815059 | -1.553941894 | 0.035002486 |
| KEGG_PROPANOATE_METABOLISM | 27 | -0.548262279 | -1.67658373 | 0.035045627 |
| KEGG_OLFACTORY_TRANSDUCTION | 64 | -0.420669197 | -1.547075375 | 0.036587767 |
| KEGG_GLYCOLYSIS_GLUCONEOGENESIS | 55 | -0.427331038 | -1.530085521 | 0.036785685 |
| KEGG_FC_EPSILON_RI_SIGNALING_PATHWAY | 72 | 0.453636967 | 1.565958038 | 0.040723223 |

**Supplementary Figure 1 |** The arthritis profile of AA rat. (A) The right foot swelling variation of rats. (B) The arthritis scores variation of rats.
